# Supplementary material for: Molecular dissection of the chromosome partitioning protein RocS and regulation by phosphorylation
Source: J Bacteriol. 2024 Sep 24;206(10):e00291-24. doi: 10.1128/jb.00291-24 (PMC11500499; doi:10.1128/jb.00291-24)

SUPPLEMENTAL MATERIAL

Molecular dissection of the chromosome partitioning protein RocS and regulation by phosphorylation

Demuysere Margaux[a], Ducret Adrien[a] and Grangeasse Christophe[a,#]

[a] Molecular Microbiology and Structural Biochemistry, UMR 5086, Université de Lyon, CNRS, Lyon, France.

Demuysere Margaux and Ducret Adrien contributed equally to this work. Author order was determined alphabetically.

[#] Correspondence should be addressed to Christophe Grangeasse christophe.grangeasse@cnrs.fr

Table S1 to Table S2

Figure S1 to Figure S8

Table S1: Strains and plasmids used in this study

Number	Strain or plasmid	Genotype	Source	Parent strain	Construction (primers in table S2)
			S. pneumoniae		
NA	R800	R6 derivative	Gift from J.-P. Claverys (France)		
Spn5	WT	R800 *rpsL1*	Gift from J.-P. Claverys (France)		
Spn1556	*ΔrocS::Janus*	R800 *rpsL1, ΔrocS::kan-rpsL*	(1)		
Spn1557	*ΔrocS*	R800 *rpsL1, ΔrocS*	(1)		
Spn3841	RocS-AH2x	R800 *rpsL1, rocS-AH2x*	This study	Spn1556	PCR 1113-1116 (PCR 1113-4910 #Spn5 + PCR 4909-1116 #Spn5)
Spn4027	RocS-AH::TM	R800 *rpsL1*, *rocS-AH::TM-Ex1MapZ*	This study	Spn1556	PCR 1113-4829#Spn5 + PCR 3686-1828#Spn3452 + PCR 5033-1116#Spn5)
Spn366	RocS-ΔAH	R800 *rpsL1, rocS- ΔAH*	(1)		
Spn4097	RocS-ΔCC1	R800 *rpsL1, rocS- ΔCC1*	This study	Spn1556	PCR 1113-1116 (PCR 1113-5054#Spn5 + PCR 5055-1116#Spn5)
Spn3913	RocS-ΔDUF	R800 *rpsL1, rocS- ΔDUF*	This study	Spn1556	PCR 1113-1116 (PCR 1113-4968#Spn5 + PCR 4967-1116#Spn5)
Spn4316	RocS-DUFQ2E	R800 *rpsL1, rocS- DUFQ2E*	This study	Spn1556	PCR 1113-1116 (PCR 1113-5183#Spn5 + PCR 5182-1116#Spn5)
Spn4317	RocS-DUFQ3E	R800 *rpsL1, rocS- DUFQ3E*	This study	Spn1556	PCR 1113-1116 (PCR 1113-5180#Spn5 + PCR 5179-1116#Spn4316)
Spn4320	RocS-DUFQ2A	R800 *rpsL1, rocS- DUFQ2A*	This study	Spn1556	PCR 1113-1116 (PCR 1113-5184#Spn5 + PCR 5182-1116#Spn5)
Spn4321	RocS-DUFQ3A	R800 *rpsL1, rocS- DUFQ3A*	This study	Spn1556	PCR 1113-1116 (PCR 1113-5181#Spn5 + PCR 5179-1116#Spn4320)
Spn2844	RocS T41D	R800 *rpsL1, rocS T41D*	This study	Spn1556	PCR 1113-1116 (PCR 1113-3999#Spn5 + PCR 2957-1116#Spn5)
Spn3840	RocS-ΔwHTH	R800 *rpsL1, rocS- ΔwHTH*	This study	Spn1556	PCR 1113-1116 (PCR 1113-4810 #Spn5 + PCR 4809-1116 #Spn5)
Spn1150	WT, HlpA-mKate2	R800 *rpsL1*, (*hlpA+*) *hlpA-mKate2-cmR*	(1)		
Spn1151	*ΔrocS*, HlpA-mKate2	*R800 rpsL1, ΔrocS, (hlpA+) hlpA-mKate2-cmR*	(1)		
Spn3880	RocS-AH2x, HlpA-mKate2	*R800 rpsL1, rocS-AH2x, (hlpA+) hlpA-mKate2-cmR*	This study	Spn3841	PCR 1113-1116 (PCR 1113-4910 #Spn5 + PCR 4909-1116 #Spn5)
Spn4027	RocS-AH::TM, HlpA-mKate2	R800 *rpsL1*, *rocS-AH::TM-Ex1MapZ, (hlpA+) hlpA-mKate2-cmR*	This study	Spn4027	PCR 1113-4829#Spn5 + PCR 3686-1828#Spn3452 + PCR 5033-1116#Spn5)
Spn1152	RocS-ΔAH, HlpA-mKate2	*R800 rpsL1, rocS- ΔAH, (hlpA+) hlpA-mKate2-cmR*	This study	Spn366	PCR 2204-2199#Spn1150
Spn4098	RocS-ΔCC1, HlpA-mKate2	*R800 rpsL1, rocS- ΔCC1, (hlpA+) hlpA-mKate2-cmR*	This study	Spn4097	PCR 2204-2199#Spn1150

Spn3913	RocS-ΔDUF, HlpA-mKate2	*R800 rpsL1, rocS- ΔDUF, (hlpA+) hlpA-mKate2-cmR*	This study	Spn3913	PCR 2204-2199#Spn1150
Spn4318	RocS-DUFQ3E, HlpA-mKate2	*R800 rpsL1, rocS- DUFQ3E, (hlpA+) hlpA-mKate2-cmR*	This study	Spn4317	PCR 2204-2199#Spn1150
Spn4322	RocS-DUFQ3A, HlpA-mKate2	*R800 rpsL1, rocS- DUFQ3A, (hlpA+) hlpA-mKate2-cmR*	This study	Spn4321	PCR 2204-2199#Spn1150
Spn2844	RocS T41D, HlpA-mKate2	*R800 rpsL1, rocS T41D, (hlpA+) hlpA-mKate2-cmR*	This study	Spn2844	PCR 2204-2199#Spn1150
Spn3840	RocS-ΔwHTH, HlpA-mKate2	*R800 rpsL1, rocS- ΔwHTH, (hlpA+) hlpA-mKate2-cmR*	This study	Spn3840	PCR 2204-2199#Spn1150
Spn1024	GFP-RocS	R800 *rpsL1, mGfp-l1-rocS*	(1)		
Spn3912	GFP-RocS-AH2x	R800 *rpsL1, mGfp-l1-rocS-AH2x*	This study	Spn1556	PCR 1113-1116 (PCR 1113-4910#Spn1024 + PCR 4904-1116#Spn5)
Spn4029	GFP-RocS-AH::TM	R800 *rpsL1*, *mGfp-l1-rocS-AH::TM-Ex1MapZ*	This study	Spn1556	PCR 1113-1116 (PCR 1113-1235#Spn1024 + PCR 2957-1116#Spn4027)
Spn1071	GFP-RocS-ΔAH	R800 *rpsL1, mGfp-l1-rocS-ΔAH*	(1)		
Spn4099	GFP-RocS-ΔCC1	R800 *rpsL1, mGfp-l1-rocS-ΔCC1*	This study	Spn1556	PCR 1113-1116 (PCR 1113-5054#Spn1024 + PCR 2957-1116#Spn3913)
Spn3915	GFP-RocS-ΔDUF	R800 *rpsL1, mGfp-l1-rocS-ΔDUF*	This study	Spn1556	PCR 1113-1116 (PCR 1113-4968#Spn1024 + PCR 4967-1116#Spn5)
Spn4319	GFP-RocS-DUFQ3E	R800 *rpsL1, mGfp-l1-rocS-DUFQ3E*	This study	Spn1556	PCR 1113-1116 (PCR 1113-5180#Spn1024 + PCR 5179-1116#Spn4317)
Spn4323	GFP-RocS-DUFQ3A	R800 *rpsL1, mGfp-l1-rocS-DUFQ3A*	This study	Spn1556	PCR 1113-1116 (PCR 1113-5181#Spn1024 + PCR 5179-1116#Spn4321)
Spn2889	GFP-RocS T41D	R800 *rpsL1, mGfp-l1-rocS T41D*	This study	Spn1556	PCR1113-1116 (PCR 1113-1568#Spn1024 + PCR 1988-1116#Spn2844)
Spn3922	GFP-RocS-ΔwHTH	R800 *rpsL1, mGfp-l1-rocS-ΔwHTH*	This study	Spn1556	PCR 1113-1116 (PCR 1113-1568 #Spn1024 + PCR 4904-1116 #Spn5)
Spn1010	RocS-pComX-Janus	*R800 ΔIS1167::P1::PcomR-comR, cpsN-O::PcomX-kan-rpsL*	(1)		
Spn1115	pComX-GFP-RocS, ΔrocS	*R800 rpsL1, ΔIS1167::P1::PcomR::comR, cpsN-O::PcomX-mGfp-rocS, ΔrocS*	(1)		
Spn2999	pComX-GFP-(AHRocS)1	*R800 rpsL1, ΔIS1167::P1::PcomR-comR, cpsN-O::PcomX-mGfp-l2-(AHrocS)1*	This study	Spn1010	PCR 1943-1946 (PCR 1946-3545 #Spn1115 + PCR 4158-1943 #Spn1115)
Spn3830	pComX-GFP-(AHRocS)2	*R800 rpsL1, ΔIS1167::P1::PcomR::comR, cpsN-O::PcomX-mGfp-l2-(AHrocS)2*	This study	Spn1010	PCR 1946-1943 (PCR 1946-4910#Spn2999 + PCR 4909-1943#Spn2999)
Spn2929	pComX-GFP-RocS	*R800 rpsL1, ΔIS1167::P1::PcomR::comR, cpsN-O::PcomX-mGfp-rocS*	This study	Spn1010	PCR1946-1943#Spn1115

Spn3337	pComX-GFP-RocS-ΔAH	*R800 rpsL1, ΔIS1167::P1::PcomR::comR, cpsN-O::PcomX-mGfp-rocS-ΔAH*	This study	Spn1010	PCR 1946-1943 (PCR 1946-2954 #Spn2929 + PCR 2082-1943 #Spn1010)
Spn3760	pComX-GFP-RocS-ΔAH, HlpA-mKate2	*R800 rpsL1, ΔIS1167::P1::PcomR::comR, cpsN-O::PcomX-mGfp-rocS-ΔAH, (hlpA+) hlpA-mKate2-cm*	This study	Spn3337	PCR 2204-2199#Spn1150
Spn2927	pComX-GFP-RocS T41D	*R800 rpsL1, ΔIS1167::P1::PcomR::comR, cpsN-O::PcomX-mGfp-rocS T41D*	This study	Spn1010	PCR 1946-1943 (PCR 1946-1568#Spn1641 + PCR 3186-1235 #Spn2889 + PCR 2957-1943#Spn1113)
Spn3339	pComX-GFP-RocS T41D-ΔAH	*R800 rpsL1, ΔIS1167::P1::PcomR::comR, cpsN-O::PcomX-mGfp-rocS T41D-ΔAH*	This study	Spn1010	PCR 1946-1943 (PCR 1946-2954 #Spn2927 + PCR 2082-1943 #Spn1010)
Spn3762	pComX-GFP-RocS T41D-ΔAH, HlpA-mKate2	*R800 rpsL1, ΔIS1167::P1::PcomR::comR, cpsN-O::PcomX-mGfp-rocS T41D-ΔAH, (hlpA+) hlpA-mKate2-cm*	This study	Spn3339	PCR 2204-2199#Spn1150
			E. coli		
	XL1-Blue	*supE44 hsdR17 recA1 endA1 gyrA46 thi relA1 lac-F'[proAB + lacIqlacZΔM15 Tn10 (TcR)]*	Gift from O. Letourneur (France)		
	BTH101	*F-, cya-99, araD139, galE15, galK16, rpsL1 (StrR), hsdR2, mcrA1, mcrB1*	Euromedex		
	BL21 Star (DE3)	*F– ompT gal dcm lon hsdSB(rB–mB–) λ (DE3[lacI lacUV5-T7p07 ind1 sam7 nin5]) [malB+]K-12(λS)*	Invitrogen		
			Plasmids		
Ec0822	pkT25	*Plac-T25- kan pACYC ori*	(2)		
Ec0825	pUT18C	*Plac-T18-, bla*	(2)		
Ec0824	pKT25-zip	*Plac-T25-leucine zipper region from yeast GCN4. kan pACYC ori*	(2)		
Ec0827	pUT18C-zip	*Plac-T18-leucine zipper region from yeast GCN4. bla*	(2)		
Ec1380	pUT18C-RocS	*Plac-T18-rocS bla*	This study		XbaI and Acc65I-digested PCR 1969a-1970#Spn5 + PCR 3488-3489#Ec0825
Ec1381	pkT25-RocS	*Plac-T25-rocS kan pACYC ori*	This study		XbaI and Acc65I-digested PCR 1969a-1970#Spn5 + PCR 3488-3489#Ec0822
Ec1972	pUT18C-RocS-ΔCC1	*Plac-T18-rocS-ΔCC1 bla*	This study	1380	Gibson assembly PCR 5054-5055#Ec1380
Ec1973	pkT25-RocS-ΔCC1	*Plac-T25-rocS-ΔCC1 kan pACYC ori*	This study	1381	Gibson assembly 5054-5055#Ec1381
Ec1974	pUT18C-RocS-ΔDUF	*Plac-T18-rocS-ΔDUF bla*	This study	1380	Gibson assembly PCR 4968-4967#Ec1380

Ec1975	pkT25-RocS-ΔDUF	*Plac-T25-rocS-ΔDUF kan pACYC ori*	This study	1381	Gibson assembly PCR 4968-4967#Ec1381
Ec1436	pt7.7-RocS-ΔAH-6His	pT7.7 derivative, encoding rocS from M1 to Q150 followed by a TEV-cleavable 6xHis tag	(1)		
Ec1556	pt7.7-RocS G15P-ΔAH-6His	pT7.7 derivative, encoding *rocS* with a G15P mutation from M1 to Q150 followed by a TEV-cleavable 6xHis tag	(1)		
Ec1856	pt7.7-RocS T41D-ΔAH-6His	pT7.7 derivative, encoding *rocS* with a T41D mutation from M1 to Q150 followed by a TEV-cleavable 6xHis tag	This study	Ec1436	Gibson assembly PCR 2957-3999#Ec1436

Table S2: Primers used in this study

Number	Name	Sequence 5'-3
1113	Fwd upstream of *rocS*	GTCTGCTATGAGTGTGGCGATTTTGGC
1116	Rev downstream of *rocS*	CTACTTTCTGTCTCTAACAATTCCCTAG
1235	Rev *rocS-ΔAH*	AGTGGATTGGACTTCTTCTTTTGC
1568	Rev *gfp-l1*	TCCGGATCCCTCGAGTTTATACAATTCATCCATACCATGTG
1828	Rev *ex1-mapZ*	ACTTCTAGTCTCATTTGAACTACTTGG
1943	Rev *cpsN*	CATCGGAACCTATACTCTTTTAG
1946	Fwd *cpsO*	ATAACAAATCCAGTAGCTTTGG
1969a	Fwd XbaI-*rocS*	TATTCTAGAGATGAGTATTGAAATGACCG
1970	Rev *rocS*-Acc65I	TATGGTACCGCTTATCCTCCAAATAAACGAGC
1988	Fwd *gfp-rocS*	CTCGAGGGATCCGGAATGAGTATTGAAATGACC
2082	Fwd *cpsN*	TTTCTAATATGTAACTCTTCCCAAT
2199	Fwd upstream of *hlpA*	CGAAGTTAGCTCAAGAAG
2204	Rev downstream of *hlpA*	CAGGTTGATATTATCG
2954	Fwd *cpsN-rocS*	GGGAAGAGTTACATATTGAAATTAAGTGGATTGGACTTCTTC
2957	Fwd *rocS V42*	CAGTTGTTACCAGAAGTGGCTTGATTAAGC
3186	Fwd *gfp*	ATTTCTAAAGGTGAAGAATTGTTTACA
3488	Rev XbaI pUT18C	CTCTAGAGTCGACCTGCAG
3489	Fwd Acc65I pUT18C	CGGGTACCGAGCTCGAATTCA
3490	Rev XbaI pkT25	CTCTAGAGTCGACCCTGCAG
3491	Fwd Acc65I pkT25	CGGGTACCTAAGTAACTAAGAATTC
3545	Rev *gfp-l2*	CTGCAGGAACTCGATGTCTAGTTTTTTATACAATTCATCCATACCATGTG
3686	Fwd *l1*	CTCGAGGGATCCGGA
3999	Rev *rocS T41D*	CAAGCCACTTCTGGTAACAACATCTACCCCTTTGTCA
4158	Fwd *gfp-l2-AHRocS*	CTAGACATCGAGTTCCTGCAGAAAAAAGGCTTTTTTGCTC
4809	Fwd *ΔwHTH-rocS*	CGTTTCAGAGGATATTATGAAAAAAACGATTTTTGAAGATG
4810	Rev *ΔwHTH-rocS*	AATATCCTCTGAAACGTTTTCTAGC
4829	Rev *rocS-ΔAH-l1*	TCCGGATCCCTCGAGAGTGGATTGGACTTCTTCTTTTGC
4904	Fwd *gfp-l1-ΔwHTH*	ATAAACTCGAGGGATCCGGAAAAAAAAACGATTTTTGAAG
4909	Fwd *AH-GGS-AH*	CGTTTATTTGGAGGAGGAGGAAGTAAAAAAGGCTTTTTTGC
4910	Rev *AH-GGS-AH*	GCAAAAAAGCCTTTTTTACTTCCTCCTCCTCCAAATAAACGAGC

4967	Fwd *rocS-ΔDUF*	GAAGAAGTCCAATCCAC
4968	Rev *rocS-ΔDUF*	AGTGGATTGGACTTCTTCTTTTTCTGATAACTGACG
5033	Fwd *rocS-AH::TM-Ex1MapZ*	CAAATGAGACTAGAAGTTAATCAAGGAGCTGTTTAGGTTAAATGC
5054	Fwd *rocS-ΔCC1*	TTCACTGACAGGTTCATCTTC
5055	Rev *rocS-ΔCC1*	AAGATGAACCTGTCAGTGAAAAAGACGAGCAGATGCGTATC
5179	Fwd *rocS M107*	GATGCGTATCAAAGACCGTC
5180	Rev *rocS Q106E*	GGTCTTTGATACGCATCTCCTCGTCTTTTTCTGATAACTG
5181	Rev *rocS Q106A*	GGTCTTTGATACGCATCGCCTCGTCTTTTTCTGATAACTG
5182	Fwd *rocS Q126*	CAATTGACCCTTCAAGCCATGAAGG
5183	Rev *rocSQ124EQ125E*	GGCTTGAAGGGTCAATTGTTCTTCCTGATCTAATTGTTTGTCTTTCTC
5184	Rev *rocSQ124AQ125A*	GGCTTGAAGGGTCAATTGTGCTGCCTGATCTAATTGTTTGTCTTTCTC

References

1. Mercy C, Ducret A, Slager J, Lavergne J-P, Freton C, Nagarajan SN, Garcia PS, Noirot-Gros M-F, Dubarry N, Nourikyan J, Veening J-W, Grangeasse C. 2019. RocS drives chromosome segregation and nucleoid protection in Streptococcus pneumoniae. 10. Nat Microbiol 4:1661–1670.

2. Karimova G, Ullmann A, Ladant D. 2001. Protein-protein interaction between Bacillus stearothermophilus tyrosyl-tRNA synthetase subdomains revealed by a bacterial two-hybrid system. J Mol Microbiol Biotechnol 3:73–82.

Figure S1: Alignment of the 11 C-terminal amino acids from MinD, RocS and Par displayed with Jalview. The sequences are labeled with the UniProt identifier followed by the protein name. The amino acids are colored according to the Clustalx default color scheme.

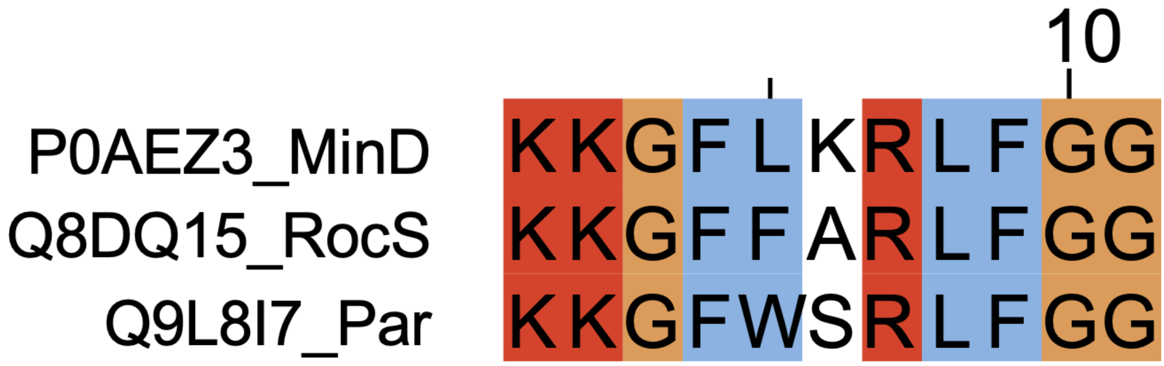

Figure S2 Western immunoblot of whole-cell lysates from *S. pneumoniae* R6 WT and *rocS::gfp-rocS mutants* strains grown to exponential phase in C+Y medium. 20 µg of total protein extracts were loaded in each lane and the expression and stability of each mutant was probed using a polyclonal anti-gfp antibody.

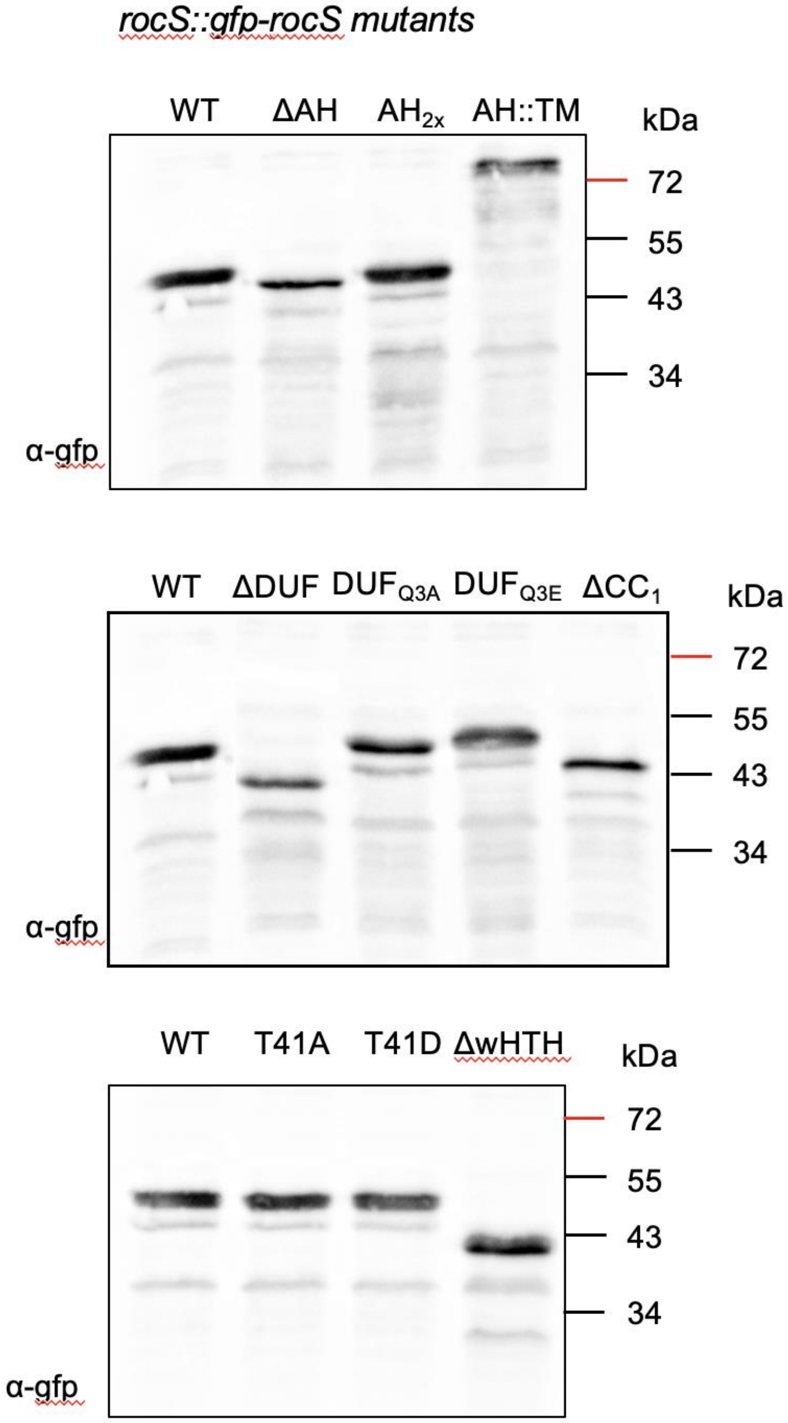

Figure S3 Representative images of cells with missegregated DNA (white arrows) from the *S. pneumoniae* R6 Δ*rocS, (hlpA+) hlpA*-*mKate2* strain. From left to right: no signal, cytosolic signal, asymmetric distribution. Scale bar, 1µm.

ΔrocS

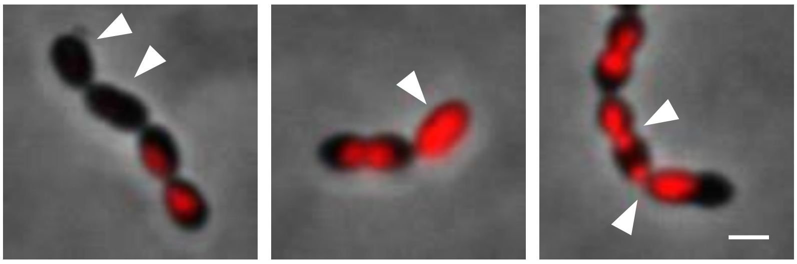

Figure S4 Coiled-coil prediction by the bioinformatic tool CoCoPRED. The sequence of full-length RocS was submitted for analysis. Upper panel: predicted propensity of a given RocS region to form a coiled-coil. The arrows indicate the limiting residues of the unique predicted CCD. A scaled representation of RocS domains has been added on the X axis to help visualize the position of the predicted CCD. The domains are color-coded according to the schematic of RocS in Fig. 1A. Lower panel: oligomeric state propensity predicted for the unique predicted coiled-coil (CCD1).

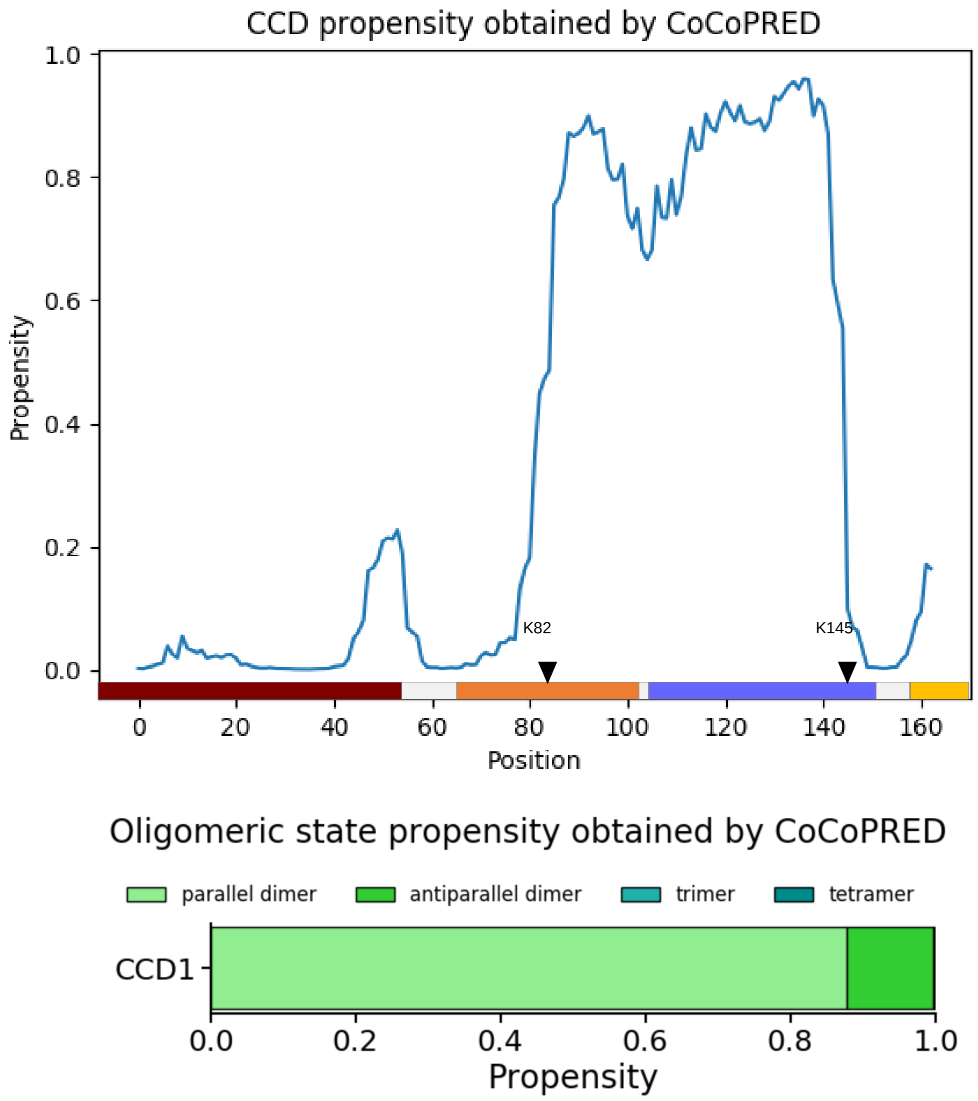

Figure S5 DUF 536 signature motif defined by Pfam HMM models and retrieved from InterPro https://www.ebi.ac.uk/interpro/entry/pfam/PF04394/logo/.

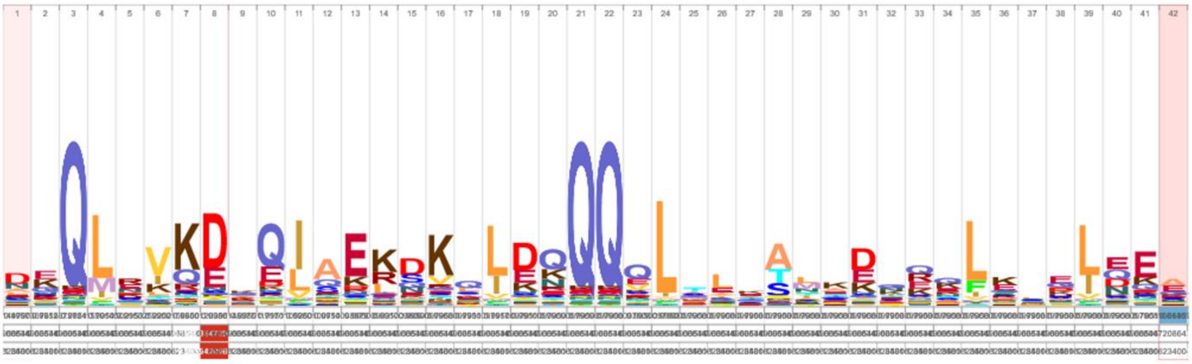

Figure S6 Gel-filtration and SDS-PAGE analysis of RocS-T41D-ΔAH. Following a nickel-affinity purification, the recombinant protein was subjected to dialysis and cleavage by the tobacco Etch virus (TEV) protease to remove the affinity tag. A second purification step on a nickel-affinity column enabled the recovery of cleaved protein, which was further separated from contaminants *via* gel filtration. The peak (left panel) was analyzed on a 12.5% SDS-PAGE stained by Coomassie (right panel).

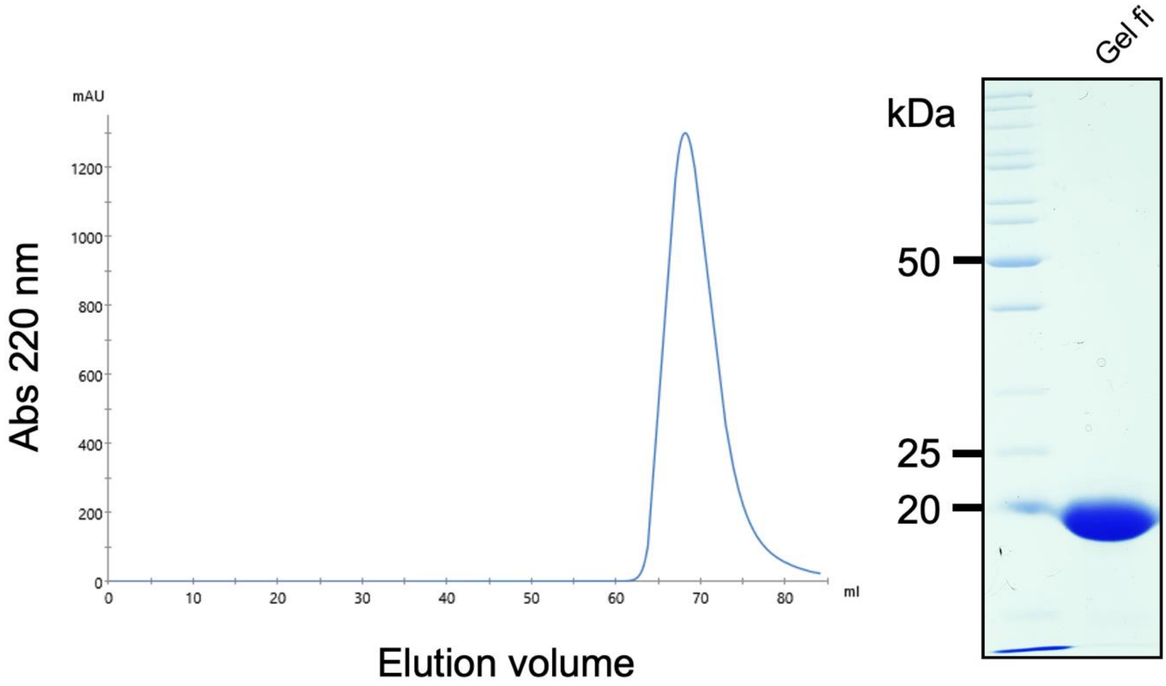

Figure S7 Bacterial two-hybrid assay. The self-interaction between RocS, RocS-T41D and RocS-ΔwHTH were assessed after co-transformation of the T18- and T25-constructs in *E. coli* BTH101 and incubation at room temperature for 48h. A blue coloration indicates a positive interaction.

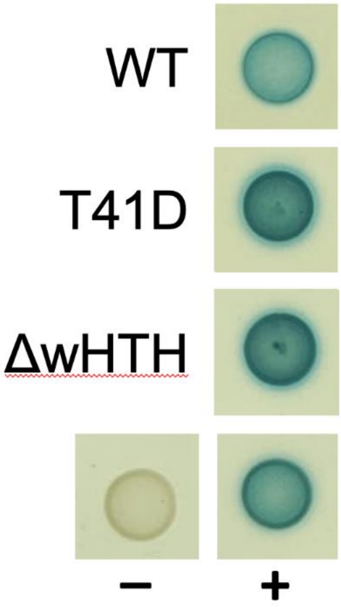

Figure S8 Distribution of the C-terminal sequences of DUF536 containing proteins that are predicted to form an amphipathic helix. The C-terminal sequences from the 892 proteins containing a DUF 536 domain were retrieved and used to reconstruct a phylogenetic tree. The C-terminal sequences predicted to form an amphipathic helix are shown in dark blue when the score is high (n=691;77.4%). The C-terminal sequences that do not form an amphipathic helix are shown in yellow (n=201;22.5%). The C-terminal sequences of RocS (Q8DQ15) and Par (Q9L8I7) are highlighted in red and in magenta respectively.

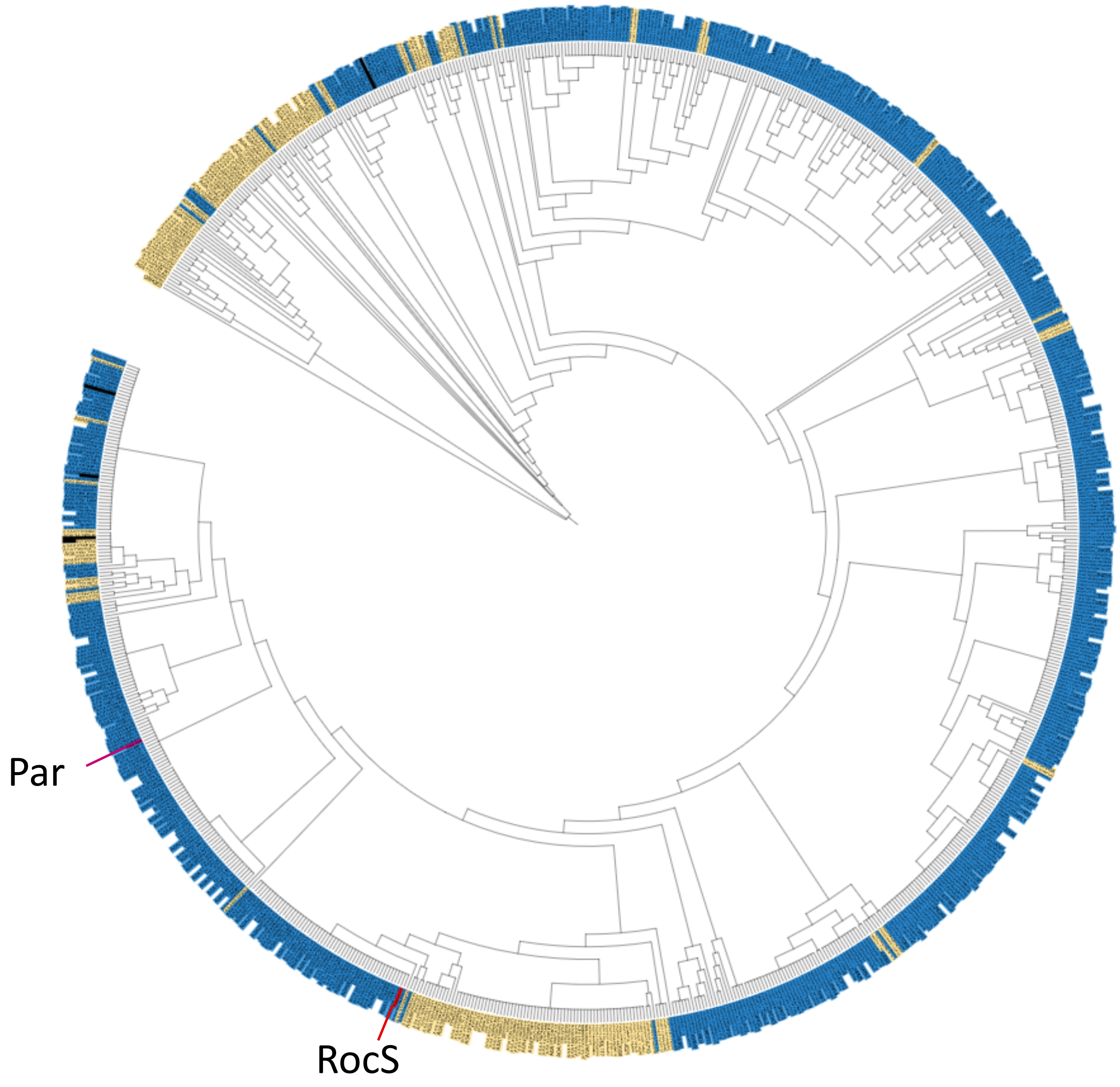

Supplement: Supplemental material — Fig. S1 to S8; Tables S1 and S2. [file jb.00291-24-s0001.pdf]
